# Temporal trend and climate factors of hemorrhagic fever with renal syndrome epidemic in Shenyang City, China

**DOI:** 10.1186/1471-2334-11-331

**Published:** 2011-12-02

**Authors:** Xiaodong Liu, Baofa Jiang, Weidong Gu, Qiyong Liu

**Affiliations:** 1 Department of Epidemiology and Health Statistics, School of Public Health, Shandong University, Shandong, PR China; 2 State Key Laboratory for Infectious Disease Prevention and Control, National Institute for Communicable Disease Control and Prevention, China CDC; 3 China CDC Key Laboratory of Surveillance and Early-Warning on Infectious Disease, Beijing, PR China; 4 Shandong University Climate Change and Health Center, Shandong, PR China; 5 Division of Infectious Diseases, William C. Gorgas Center for Geographic Medicine, University Alabama at Birmingham School of Medicine, Birmingham, USA; 6 EDEB/NCEZID/CDC, Atlanta, Georgia 30329, USA

## Abstract

**Background:**

Hemorrhagic fever with renal syndrome (HFRS) is an important infectious disease caused by different species of hantaviruses. As a rodent-borne disease with a seasonal distribution, external environmental factors including climate factors may play a significant role in its transmission. The city of Shenyang is one of the most seriously endemic areas for HFRS. Here, we characterized the dynamic temporal trend of HFRS, and identified climate-related risk factors and their roles in HFRS transmission in Shenyang, China.

**Methods:**

The annual and monthly cumulative numbers of HFRS cases from 2004 to 2009 were calculated and plotted to show the annual and seasonal fluctuation in Shenyang. Cross-correlation and autocorrelation analyses were performed to detect the lagged effect of climate factors on HFRS transmission and the autocorrelation of monthly HFRS cases. Principal component analysis was constructed by using climate data from 2004 to 2009 to extract principal components of climate factors to reduce co-linearity. The extracted principal components and autocorrelation terms of monthly HFRS cases were added into a multiple regression model called principal components regression model (PCR) to quantify the relationship between climate factors, autocorrelation terms and transmission of HFRS. The PCR model was compared to a general multiple regression model conducted only with climate factors as independent variables.

**Results:**

A distinctly declining temporal trend of annual HFRS incidence was identified. HFRS cases were reported every month, and the two peak periods occurred in spring (March to May) and winter (November to January), during which, nearly 75% of the HFRS cases were reported. Three principal components were extracted with a cumulative contribution rate of 86.06%. Component 1 represented MinRH_0_, MT_1_, RH_1_, and MWV_1_; component 2 represented RH_2_, MaxT_3_, and MAP_3_; and component 3 represented MaxT_2_, MAP_2_, and MWV_2_. The PCR model was composed of three principal components and two autocorrelation terms. The association between HFRS epidemics and climate factors was better explained in the PCR model (*F *= 446.452, *P *< 0.001, adjusted *R*^2 ^= 0.75) than in the general multiple regression model (*F *= 223.670, *P *< 0.000, adjusted *R*^2 ^= 0.51).

**Conclusion:**

The temporal distribution of HFRS in Shenyang varied in different years with a distinctly declining trend. The monthly trends of HFRS were significantly associated with local temperature, relative humidity, precipitation, air pressure, and wind velocity of the different previous months. The model conducted in this study will make HFRS surveillance simpler and the control of HFRS more targeted in Shenyang.

## Background

Hemorrhagic fever with renal syndrome (HFRS), with characteristics of fever, hemorrhage, kidney damage and hypotension, is an important infectious disease caused by different species of hantaviruses. In hantavirus-endemic areas, HFRS outbreaks have occurred among farmers and others who have close contact with excreta of infected rodents [1,2]. HFRS has been recognized as a notable public health problem in China [3]. It is considered that the number of HFRS cases in China accounts for 90% of the total cases worldwide [4,5]. At present, HFRS is endemic in 28 of 31 provinces, autonomous regions, and metropolitan areas in mainland China [6]. Although some prevention and control measures such as scientific rodent control, vaccination and environmental management have been performed, HFRS remains a serious public health problem with about 20,000-50,000 human cases annually in mainland China [7].

As a rodent-borne disease with a seasonal distribution, external environmental factors including climate factors may play a significant role in its transmission. Studies in different areas of China and other countries have suggested that climate factors, such as temperature, precipitation, and relative humidity, may influence the incidence of HFRS [8-10]. The role of climate factors in such transmission may vary, given the large variations in climate types, ecological characteristics, population immunity, public health intervention measures, and socioeconomic status in the different regions.

Liaoning Province is one of the most seriously affected areas with the highest incidence during the years 2004 and 2005 [11]. Since the first HFRS case in 1958, Shenyang, the capital city of Liaoning Province, has been among the forefront of the province in recent years, with all its 13 districts/counties being susceptible to the disease [12]. Through the use of a 6-year (2004-2009) record of HFRS cases in Shenyang, this study aimed to characterize the seasonal patterns of HFRS distribution and explore associations between climate factors and HFRS transmission.

## Methods

### Study area

**Figure 1:**
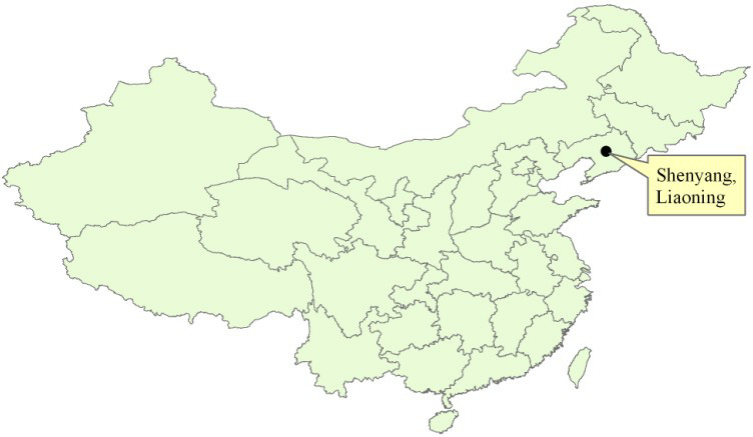
**The black dot indicates the location of Shenyang in China**.

The study area covers Shenyang, the capital city of Liaoning Province, located between latitude 41°48' and 41°50' north and longitude 123°23' and 123°40' east (Figure 1). The city comprises 9 districts and four counties with a total land area of 12,948 km^2 ^and a population of about 7,860,000 in 2009. Shenyang has a temperate climate with dry winters and wet, hot summers. The annual temperature ranges from -29 to +36°C and the annual mean temperature is about 7.5°. The annual rainfall is typically between 600 and 800 mm. HFRS is a rodent-borne disease. Therefore, rodent control is important for disease control. The major rodent species in Shenyang are *Apodemus agrarius and Rattus norvegicus*. The widespread distribution of rodents makes their effective control difficult.

## Data collection and management

### Surveillance data

In this study, records on HFRS cases from 2004 to 2009 were obtained from the National Notifiable Disease Surveillance System. The disease surveillance data used in this study was already anonymous. We state that the China CDC provides the permission to use the data. All HFRS cases were first diagnosed by clinical symptoms. Patient blood samples were collected in hospitals and sent to the laboratory of Liaoning Provincial Center for Disease Control and Prevention (CDC) for serological confirmation. Finally, the data were collected by case number according to the sampling results. There might be admission rate bias in the disease report, but this has been reduced as much as possible. According to the legislation, physicians in hospitals must report every case of HFRS to the local health authority within 12 hours. The health authority reports these cases to the next level of the organization every month. Therefore, it is believed that the degree of compliance in disease notification over the study period was consistent. Demographic data were obtained from the statistical yearbooks of Liaoning Province, which were compiled by the Liaoning Provincial Bureau of Statistics.

### Meteorological data

From 2004 to 2009, monthly climate data from approximately 700 surveillance stations in mainland China were collected from the China Meteorological Data Sharing Service System http://cdc.cma.gov.cn/index.jsp. The climate factors included monthly mean temperature (MT), monthly mean maximum temperature (MaxT), monthly mean minimum temperature (MinT), monthly mean relative humidity (RH), monthly mean minimum relative humidity (MinRH), monthly accumulative precipitation (AP), monthly mean air pressure (MAP), monthly mean wind velocity (MWV), and monthly sunshine duration (SD).

### Temporal distribution analysis

The annual HFRS incidence from 2004 to 2009 was calculated and plotted to observe annual fluctuations in Shenyang. Cumulative HFRS cases for each month from 2004 to 2009 were also calculated to observe seasonal fluctuations.

### Cross-correlation and autocorrelation analysis

A cross-correlation analysis was conducted to detect the effects of climate factors on HFRS transmission with a lag time of 6 months. The cross-correlation could be observed if the absolute value of cross-correlation coefficient (CCF) was two times larger than the standard error (SE). An autocorrelation analysis was performed to explore whether the monthly HFRS cases were affected by the cases in previous months, by using the Ljung-Box Q test, autocorrelation coefficient (AC) and partial autocorrelation coefficient (PAC).

### Multivariate time series analysis

Principal component analysis (PCA) was constructed by using climate data from 2004 to 2009 to extract principal components. The extracted principal components and autocorrelation terms of monthly HFRS cases were added into a multiple regression model called principal components regression model (PCR) to quantify the relationship between climate factors, autocorrelation terms and transmission of HFRS. The PCR model was compared to a general multiple regression model conducted with only climate factors as independent variables.

### Ethical review

The present study was reviewed by the research institutional review board of Shandong University and China CDC, and it is found that utilization of disease surveillance and meteorological data did not require oversight by an ethics committee.

## Results

### Descriptive analysis

There were 1,931 cases in total in Shenyang over the study period. HFRS cases were reported in all districts/counties of Shenyang during this period, with annual average incidences ranging from 0.15 to 34.65 per 100,000.

### Temporal distribution of HFRS in Shenyang

**Figure 2:**
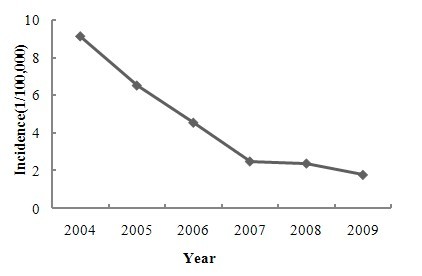
**Annual incidence of HFRS from 2004 to 2009 in Shenyang, China**.

**Figure 3:**
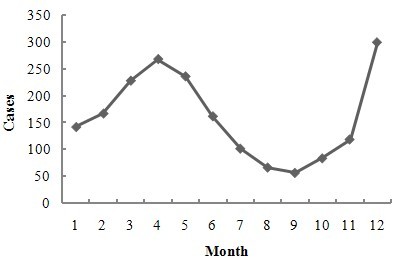
**Monthly distribution of HFRS cases in Shenyang, China**.

The annual HFRS incidence in Shenyang varied from 2004 to 2009. The highest incidence of 9.11 cases per 100,000 persons occurred in 2004, and the lowest incidence of 1.75 cases per 100,000 occurred in 2009. A distinctly declining temporal trend in annual HFRS incidence was identified (Figure 2). HFRS cases were reported every month, and the two peak periods occurred in spring (March to May) and winter (November to January), during which nearly 75% of the HFRS cases were reported (Figure 3).

### Cross-correlation between monthly HFRS incidence and climate factors

As shown in Table 1 (See additional file 1), the monthly mean relative humidity (RH_0_) and monthly mean minimum relative humidity (MinRH_0_) were negatively correlated with the monthly number of HFRS cases in Shenyang, while monthly mean wind velocity (MWV_0_) was positively correlated with the monthly number of HFRS cases in the same month. During the previous month, the monthly number of HFRS cases was negatively correlated with climate factors, including the monthly mean temperature (MT_1_), monthly mean maximum temperature (MaxT_1_), monthly mean minimum temperature (MinT_1_), monthly mean relative humidity (RH_1_), monthly accumulative precipitation (AP_1_), as well as positively correlated with monthly mean wind velocity (MWV_1_). During the previous 2 months, the monthly HFRS cases were negatively correlated with the monthly mean temperature (MT_2_), monthly mean maximum temperature (MaxT_2_), monthly mean minimum temperature (MinT_2_), and monthly mean relative humidity (RH_2_), but positively correlated with the monthly mean air pressure (MAP_2_) and monthly mean wind velocity (MWV_2_). During the previous 3 months, the monthly HFRS cases were negatively correlated with the monthly mean temperature (MT_3_), and monthly mean maximum temperature (MaxT_3_), but positively correlated with the monthly mean air pressure(MAP_3_).

### Autocorrelation of monthly number of HFRS cases

The P value of the Ljung-Box Q statistic of each lagged month was < 0.05. The absolute value of the autocorrelation coefficient and partial autocorrelation coefficient during the first two lagged months (Lag1 and Lag2) was greater than that of other lagged months, which indicated that there was a strong autocorrelation of monthly HFRS cases during the first two lagged months (Table 2, See additional file 2).

### Model evaluation

The PCA was conducted by using variables RH_0_, MinRH_0_, MWS_0_, MT_1_, MaxT_1_, MinT_1_, RH_1_, AP_1_, MWS_1_, MT_2_, MaxT_2_, MinT_2_, RH_2_, MAP_2_, MWS_2_, MT_3_, MaxT_3_, and MAP_3_. Three principal components were extracted with a cumulative contribution rate of 86.06% (Table 3, See additional file 3). The loadings of the three principal components of each variable were calculated (Table 4 See additional file 4). Component 1 represented MinRH_0_, MT_1_, RH_1_, and MWV_1_; component 2 represented RH_2_, MaxT_3_, and MAP_3_; and component 3 represented MaxT_2_, MAP_2_, and MWV_2_. Consequently, the three principal components and the two autocorrelation terms of monthly HFRS cases (Lag1, and Lag2) were added into the multiple regression model. The following model gave the best results:

Y=-0.078component1-0.168component2-0.372component3+0.395Lag1+0.378Lag2

**Figure 4:**
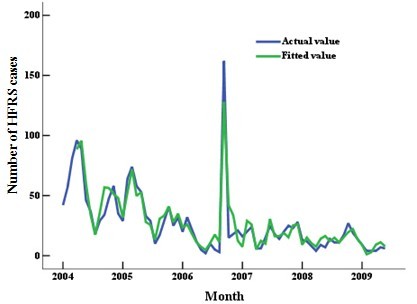
**Fitted (green) and actual (blue) monthly numbers of HFRS cases in Shenyang, China**.

The association between HFRS epidemics and climate factors was better explained in the PCR model (Figure 4, *F *= 446.452, *P *< 0.001, adjusted *R*^2 ^= 0.75) than in the general multiple regression model (*F *= 223.670, *P *< 0.000, adjusted *R*^2 ^= 0.51).

## Discussion

The results of this study confirmed that the temporal distribution in Shenyang varied by months and years, which indicated that public health resource allocations should focus on the months with the highest HFRS incidence.

Previous studies have debated the association between HFRS epidemics and temperature in areas that are severely affected by HFRS around the world; especially in many areas of China [8,9,13,14]. These studies have indicated that temperature is an important factor for HFRS epidemics in China. Some studies have found that temperature has a positive effect on HFRS [8,13], whereas a negative effect has been found in other studies [9,14,15]. The results of the present study showed that MT_1_, MaxT_2 _and MaxT_3 _were negatively associated with the monthly HFRS cases in Shenyang. Based on the results of this study, effective surveillance of HFRS epidemics can be conducted by monitoring the fluctuations of MT_1_, MaxT_2 _and MaxT_3 _in the area, and targeted countermeasures can be taken ahead of time.

Lin et al. have indicated that relative humidity might make some contribution to the high epidemic occurrence of HFRS in Liaoning Province [11]. Liu et al. have applied a case-crossover design and conditional logistic regression to analyze the relationship between incidence rates of HFRS and meteorological variables in one national surveillance area for HFRS in Shandong Province [9]. They have found that the MRH, AP, and WV were positively associated with HFRS incidence. In contrast, some researchers have reported that the associations between HFRS epidemics and precipitation and MRH are not significant [16]. The present study found that relative humidity factors, including MinRH_0_, RH_1 _and RH_2_, affected HFRS negatively, which was consistent with previous studies in China [8,14,17]. In addition, MWV_1_, MWV_2_, MAP_2 _and MAP_3 _had positive effects on the monthly HFRS cases in cross-correlation analysis, but negative effects in multiple regression, which could have been due to the combined effects with other elements of the same principal component.

Excessive precipitation could have a negative impact on rodents by destroying their habitats in Eastern China [18,19]. In addition, frequent rainfall may decrease the likelihood of rodent-to-rodent contact, rodent-to-human contact, and virus transmission due to decreased rodent activity and reduced human exposure [18]. The impact of precipitation on HFRS in this study was consistent with previous studies. Although AP_1 _was not included in the model as a predictor of HFRS, it still had a negative effect on HFRS, according to cross-correlation analysis. The absence of AP in the model was due to its relative small loading. Therefore, when forecasting the trend of HFRS in Shenyang, AP_1 _still should be taken into consideration. In addition, the monthly SD was not related to the monthly number of HFRS cases. This finding was not consistent with some other studies [9,14,17], which could have been due to different study areas. This result indicated that it is not necessary to consider SD as a predictor of HFRS in Shenyang.

In the present study, multiple regression was constructed to detect the relationship between HFRS epidemics and climate factors in Shenyang, by incorporating climate factors of previous months based on a PCA. This model was reliable and had a good fit (adjusted *R*^2 ^of fitting = 0.75). Previous studies also have used other methods to identify climate-related risk factors of HFRS, such as Bayesian discriminant analysis [17], structure equation model [20], generalized linear model, and generalized additive model [21]. To obtain the expected results, it was necessary to choose the most suitable method according to available data.

Despite the insights gained, the limitations of our study should also be acknowledged. First, this study examined only the effect of climate factors and autoregression on HFRS epidemics, without taking into account rat density, socioeconomic circumstances, and countermeasures. Transmission of hantaviruses to humans is also related to other factors such as human activities, farming patterns, and rodent abatement strategies [22]. Second, the model in this study was constructed by using relatively short-term data for a 6-year period, which could have made the results less reliable. Third, this study didn't consider the impact of the vaccination. Vaccination plays an important role in the control of hemorrhagic fever with renal syndrome. A comprehensive intervention based on vaccination has been adopted in Shenyang since 2004 [23]. Vaccination partly contributed to the obvious decline of the annual HFRS incidence in 2007 compared to that in 2006. Climatic factors also contributed to the lower incidence in 2007 compared with that in 2006, because the amount of precipitation of 2007 is much higher than that in 2006. As the vaccination intervention is done in specific areas, it is difficult to take the impact of the vaccination into the model. HFRS incidence in the past two decades was much higher in the frigid-temperate zone, mostly in northeastern China, followed by the warm-temperate zone, with a lower incidence seen in southeastern China, where there are higher temperatures, higher humidity, and greater precipitation [6,24]. This was consistent with the results of our analysis of climate factors. These results lay a foundation for HFRS surveillance and control in this area. As a next step, it will be advantageous to explore the method for assigning fluctuant coefficients to climate factors, to improve upon the current methods. Furthermore, it is necessary to conduct further research on how socioeconomic factors affect HFRS transmission in Shenyang, which will assist in amending prevention and control strategies.

## Conclusion

This study shows that the temporal distribution of HFRS in Shenyang varied in different years(2004-2009), with a distinctly declining trend. The monthly trends for HFRS were significantly associated with local temperature, relative humidity, precipitation, air pressure, and wind velocity of the previous months. The model conducted in this study will make HFRS surveillance simpler and its control more targeted in Shenyang.

## Competing interests

The authors declare that they have no competing interests.

## Authors' contributions

XL, QL, BJ were involved in the research design, execution and write up of the first draft of the manuscript. XL, QL and WG contributed to data collection and statistical analysis. All authors contributed to the writing of the manuscript and approved the submitted version of the manuscript.

## Pre-publication history

The pre-publication history for this paper can be accessed here:

http://www.biomedcentral.com/1471-2334/11/331/prepub

## Supplementary Material

Additional file 1 **Table 1**. Cross correlation between monthly HFRS cases and climate factors in Shenyang, China. Click here for file

Additional file 2 **Table 2**. Autocorrelation and partial correlation of monthly HFRS cases in Shenyang, China. Click here for file

Additional file 3 **Table 3**. Total variance of relative variables explained by PCA. Click here for file

Additional file 4 **Table 4**. PCA, matrix of component loadings. Click here for file
