# Supplementary material for: Temporal trend and climate factors of hemorrhagic fever with renal syndrome epidemic in Shenyang City, China
Source: BMC Infect Dis. 2011 Dec 2;11:331. doi: 10.1186/1471-2334-11-331 (PMC3247297; doi:10.1186/1471-2334-11-331)
Supplement: Additional file 1 — Table 1. Cross correlation between monthly HFRS cases and climate factors in Shenyang, China. [file 1471-2334-11-331-S1.DOC]

**Additional file 1**

| Lag | MAP | | MT | | MinMT | | MaxMT | | RH | | MinRH | | AP | | MWV | | SD | |
| --- | --- | --- | --- | --- | --- | --- | --- | --- | --- | --- | --- | --- | --- | --- | --- | --- | --- | --- |
|  | *CCF* | *SE* | *CCF* | *SE* | *CCF* | *SE* | *CCF* | *SE* | *CCF* | *SE* | *CCF* | *SE* | *CCF* | *SE* | *CCF* | *SE* | *CCF* | *SE* |
| -6 | -0.120 | 0.124 | 0.179 | 0.124 | 0.190 | 0.124 | 0.170 | 0.124 | 0.046 | 0.124 | 0.030 | 0.124 | 0.111 | 0.124 | -0.003 | 0.124 | -0.157 | 0.124 |
| -5 | 0.000 | 0.123 | 0.056 | 0.123 | 0.077 | 0.123 | 0.041 | 0.123 | 0.112 | 0.123 | 0.241 | 0.123 | 0.059 | 0.123 | -0.106 | 0.123 | -0.162 | 0.123 |
| -4 | 0.171 | 0.122 | -0.104 | 0.122 | -0.082 | 0.122 | -0.120 | 0.122 | 0.056 | 0.122 | 0.298 | 0.122 | 0.001 | 0.122 | -0.086 | 0.122 | -0.194 | 0.122 |
| -3 | 0.306 | 0.121 | -0.243 | 0.121 | -0.234 | 0.121 | -0.244 | 0.121 | -0.129 | 0.121 | 0.066 | 0.121 | -0.174 | 0.121 | 0.041 | 0.121 | -0.130 | 0.121 |
| -2 | 0.323 | 0.120 | -0.287 | 0.120 | -0.280 | 0.120 | -0.295 | 0.120 | -0.344 | 0.120 | -0.110 | 0.120 | -0.240 | 0.120 | 0.302 | 0.120 | -0.088 | 0.120 |
| -1 | 0.228 | 0.120 | -0.261 | 0.120 | -0.254 | 0.120 | -0.276 | 0.120 | -0.513 | 0.120 | -0.179 | 0.120 | -0.266 | 0.120 | 0.475 | 0.120 | -0.068 | 0.120 |
| 0 | 0.132 | 0.119 | -0.165 | 0.119 | -0.154 | 0.119 | -0.181 | 0.119 | -0.446 | 0.119 | -0.271 | 0.119 | -0.161 | 0.119 | 0.471 | 0.119 | -0.135 | 0.119 |
| 1 | -0.024 | 0.120 | -0.028 | 0.120 | -0.018 | 0.120 | -0.039 | 0.120 | -0.209 | 0.120 | 0.004 | 0.120 | 0.033 | 0.120 | 0.254 | 0.120 | -0.149 | 0.120 |
| 2 | -0.212 | 0.120 | 0.157 | 0.120 | 0.175 | 0.120 | 0.138 | 0.120 | -0.118 | 0.120 | 0.082 | 0.120 | 0.258 | 0.120 | 0.209 | 0.120 | -0.145 | 0.120 |
| 3 | -0.264 | 0.121 | 0.268 | 0.121 | 0.289 | 0.121 | 0.242 | 0.121 | -0.001 | 0.121 | 0.187 | 0.121 | 0.347 | 0.121 | 0.203 | 0.121 | -0.036 | 0.121 |
| 4 | -0.263 | 0.122 | 0.341 | 0.122 | 0.350 | 0.122 | 0.330 | 0.122 | -0.038 | 0.122 | 0.084 | 0.122 | 0.235 | 0.122 | 0.136 | 0.122 | 0.021 | 0.122 |
| 5 | -0.220 | 0.123 | 0.318 | 0.123 | 0.317 | 0.123 | 0.314 | 0.123 | -0.092 | 0.123 | -0.005 | 0.123 | 0.153 | 0.123 | 0.043 | 0.123 | 0.104 | 0.123 |
| 6 | -0.050 | 0.124 | 0.194 | 0.124 | 0.200 | 0.124 | 0.185 | 0.124 | -0.023 | 0.124 | 0.099 | 0.124 | -0002 | 0.124 | 0.024 | 0.124 | 0.014 | 0.124 |
