# Supplementary material for: Temporal trend and climate factors of hemorrhagic fever with renal syndrome epidemic in Shenyang City, China
Source: BMC Infect Dis. 2011 Dec 2;11:331. doi: 10.1186/1471-2334-11-331 (PMC3247297; doi:10.1186/1471-2334-11-331)
Supplement: Additional file 2 — Table 2. Autocorrelation and partial correlation of monthly HFRS cases in Shenyang, China. [file 1471-2334-11-331-S2.DOC]

**Additional file 2**

| Lag | *AC* | *PAC* | *LB* | *P* |
| --- | --- | --- | --- | --- |
| 1 | 0.421 | 0.421 | 14.834 | <0.001 |
| 2 | 0.303 | 0.153 | 23.476 | <0.001 |
| 3 | 0.209 | 0.046 | 27.703 | <0.001 |
| 4 | 0.106 | -0.035 | 28.864 | <0.001 |
| 5 | 0.092 | 0.029 | 29.893 | <0.001 |
| 6 | 0.126 | 0.086 | 31.684 | <0.001 |
| 7 | 0.113 | 0.033 | 32.989 | <0.001 |
| 8 | 0.160 | 0.087 | 35.294 | <0.001 |
| 9 | 0.142 | 0.027 | 37.480 | <0.001 |
| 10 | 0.228 | 0.151 | 42.763 | <0.001 |
| 11 | 0.230 | 0.081 | 47.872 | <0.001 |
| 12 | 0.284 | 0.140 | 54.567 | <0.001 |
