# Supplementary material for: Temporal trend and climate factors of hemorrhagic fever with renal syndrome epidemic in Shenyang City, China
Source: BMC Infect Dis. 2011 Dec 2;11:331. doi: 10.1186/1471-2334-11-331 (PMC3247297; doi:10.1186/1471-2334-11-331)
Supplement: Additional file 3 — Table 3. Total variance of relative variables explained by PCA. [file 1471-2334-11-331-S3.DOC]

**Additional file 3**

| Component | Initial eigenvalues | | | Extraction sums of squared loadings | | |
| --- | --- | --- | --- | --- | --- | --- |
|  | Total | % of variance | Cumulative % | Total | % of variance | Cumulative % |
| 1 | 9.737 | 54.095 | 54.095 | 9.737 | 54.095 | 54.095 |
| 2 | 4.324 | 24.021 | 78.116 | 4.324 | 24.021 | 78.116 |
| 3 | 1.431 | 7.948 | 86.064 | 1.431 | 7.948 | 86.064 |
| 4 | 0.969 | 5.384 | 91.449 |  |  |  |
| 5 | 0.373 | 2.072 | 93.521 |  |  |  |
| 6 | 0.333 | 1.848 | 95.369 |  |  |  |
| 7 | 0.263 | 1.459 | 96.828 |  |  |  |
| 8 | 0.200 | 1.111 | 97.939 |  |  |  |
| 9 | 0.124 | 0.689 | 98.628 |  |  |  |
| 10 | 0.087 | 0.481 | 99.109 |  |  |  |
| 11 | 0.082 | 0.454 | 99.563 |  |  |  |
| 12 | 0.054 | 0.300 | 99.863 |  |  |  |
| 13 | 0.014 | 0.080 | 99.943 |  |  |  |
| 14 | 0.005 | 0.030 | 99.973 |  |  |  |
| 15 | 0.004 | 0.020 | 99.994 |  |  |  |
| 16 | 0.001 | 0.005 | 99.999 |  |  |  |
| 17 | 0.000 | 0.001 | 100.000 |  |  |  |
| 18 | 0.000 | 0.000 | 100.000 |  |  |  |
