# Supplementary material for: Temporal trend and climate factors of hemorrhagic fever with renal syndrome epidemic in Shenyang City, China
Source: BMC Infect Dis. 2011 Dec 2;11:331. doi: 10.1186/1471-2334-11-331 (PMC3247297; doi:10.1186/1471-2334-11-331)
Supplement: Additional file 4 — Table 4. PCA, matrix of component loadings. [file 1471-2334-11-331-S4.DOC]

**Additional file4**

| Variable | Component | | |
| --- | --- | --- | --- |
|  | 1 | 2 | 3 |
| RH_0_ | -0.854 | -0.402 | 0.026 |
| MinRH_0_ | -0.911 | 0.079 | 0.294 |
| MWV_0_ | 0.720 | 0.640 | 0.072 |
| MT_1_ | 0.960 | 0.157 | -0.199 |
| MaxT_1_ | 0.847 | -0.329 | -0.337 |
| MinT_1_ | 0.735 | 0.627 | 0.084 |
| RH_1_ | 0.960 | 0.153 | -0.201 |
| AP_1_ | 0.716 | 0.634 | 0.059 |
| MWV_1_ | 0.957 | 0.139 | -0.201 |
| MT_2_ | 0.838 | -0.346 | -0.333 |
| MaxT_2_ | 0.731 | -0.319 | 0.415 |
| MinT_2_ | 0.580 | -0.651 | 0.232 |
| RH_2_ | 0.283 | -0.730 | -0.242 |
| MAP_2_ | 0.576 | -0.240 | 0.582 |
| MWV_2_ | 0.669 | 0.242 | 0.533 |
| MT_3_ | -0.706 | 0.421 | -0.198 |
| MaxT_3_ | -0.438 | 0.781 | -0.181 |
| MAP_3_ | -0.092 | 0.833 | 0.068 |
